# Eco-friendly extension of postharvest longevity in Alstroemeria cut flowers using melatonin and putrescine treatments

**DOI:** 10.1016/j.heliyon.2025.e42343

**Published:** 2025-01-29

**Authors:** Negin Hosseini, Zohreh Jabbarzadeh, Jafar Amiri

**Affiliations:** Department of Horticultural Science, Faculty of Agriculture, Urmia University, Urmia, Iran

**Keywords:** Alstroemeria, Cut flowers, Phenylalanine ammonia-lyase activity, Postharvest quality, Vase life, Water retention

## Abstract

Postharvest longevity is the most important factor concerning the commercial value of cut flowers. Alstroemeria 'Amatista' is one of the most valued species for its ornamental appeal and it has been often reported to suffer from premature senescence. The present study was undertaken to establish the efficacy of melatonin (100 and 200 μM) and putrescine (1.5 and 3 mM) as enviro-friendly compounds applied into the vase solution in extending the vase life of cut flowers of Alstroemeria by assessing different physiological parameters. Results showed that melatonin at 100 and 200 μM and putrescine at 1.5 mM significantly extended the flower vase life from 12 to 13 days (control) to 20–21 days. More precisely, both melatonin and putrescine lowered electrolyte leakage (EL) and increased relative water content (RWC), relative fresh weight (RFW), and relative solution uptake (RSU) of flowers. In addition, both compounds enhanced the total anthocyanin content, phenylalanine ammonia-lyase (PAL) and polyphenol oxidase (PPO) activities, and reduced malondialdehyde (MDA) and hydrogen peroxide (H₂O₂) content, which implies the decline in oxidative stress. These results confirm that both melatonin and putrescine act as effective and environmental-friendly exogenous applications to improve postharvest quality and extend the vase life of Alstroemeria cut flowers. The study provides important information for possible applications in the floriculture industry.

## Highlights

• Melatonin (100 and 200 μM) and putrescine (1.5 mM) significantly extended the vase life of Alstroemeria cut flowers.
• Higher concentrations of putrescine (3 mM) did not improve vase life.
• Melatonin reduced oxidative stress and maintained membrane integrity.
• Putrescine delayed senescence by stabilizing membranes, improving flower quality and longevity.

## Graphical abstract

**Figure:**
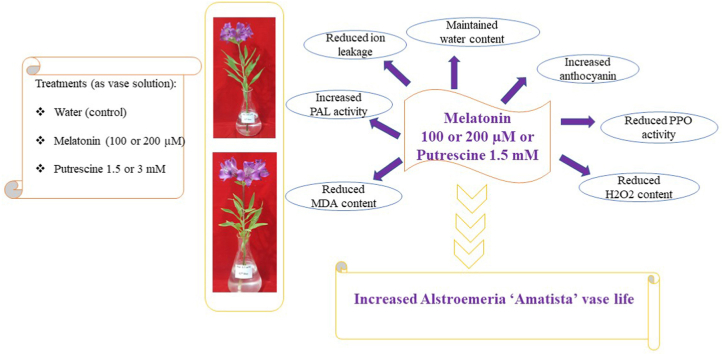

## 1 Introduction

Perennial flowering Alstroemeria (*Alstroemeria* spp.) of the family Alstroemeriaceae is an ornamental plant admired for its showy, multi-colored flowers and long flowering season [1]. Featuring these trumpet-shaped flowers, it has gained popularity in the floristry industry, from potted uses to garden and flower arrangements. The beauty, wide color range, and long flowering period from late spring to autumn have established Alstroemeria as one of the most popular cut flowers in the world. Besides aesthetic value, Alstroemeria is of economic importance due to its high production potential and adaptability to a wide range of climates, making it one of the staple crops for floriculture. However, despite the commercial importance, postharvest longevity has remained one of the big challenges facing Alstroemeria cut flowers since they always undergo early senescence, thus shortening vase life and their marketability [2]. Because of its relatively low temperature requirement for growth, 13–16 °C, and adaptability to different global regions, the production potential of Alstroemeria as a cut flower is very high [3].

Melatonin is a low-molecular-weight indoleamine that is widely distributed among organisms, from bacteria and mammals to plants. Its content is highest in cell membranes, followed by mitochondria, nuclei, and cytoplasm. Melatonin synthesis was primarily found to take place in mitochondria and chloroplasts. In plants, it has been recognized to act as an agent of protection against oxidative stress [4]. In cut flowers, exogenous melatonin has been shown to extend the vase life significantly by influencing several physiological parameters. It reduces hydrogen peroxide levels, thus alleviating oxidative stress, as observed in cut roses 'Red First' [5]. Melatonin also reduces malondialdehyde concentrations, which is a key indicator of lipid peroxidation and cellular damage, with positive effects noted in tuberose flowers [6]. By maintaining relative water content, melatonin maintains freshness and extends flower longevity of cut roses and carnations ([5,7]). On the other hand, melatonin maintains cellular integrity through the stabilization of membrane structure, which delays senescence, observed in 'Bltico' carnations [7]. Finally, melatonin increases the levels of bioactive compounds and antioxidant activity, promoting further delay in senescence, as observed in studies on carnations [7].

Organic polycations, polyamines are variable hydrocarbon chains with multiple amino groups. Common examples are the diamines such as putrescine, triamines such as spermidine, and tetraamines such as spermine [8]. Based on classification according to amine groups, putrescine is a type of diamine, while spermidine and spermine are triamine and tetraamine polyamines, respectively, meaning from putrescine onwards to spermidine and spermine, there is an increase in amine content [9]. Of them, putrescine is widely reported to improve vase life in cut flowers through its mode of action by various physiological changes. It maintains cell membrane integrity and acts as an anti-senescence factor, delaying tissue breakdown in cut flowers [10]. In addition, putrescine improves the water relations of cut flowers, which is one of the most important factors responsible for maintaining quality and prolonging the vase life of flowers [11]. It also promotes an increase in fresh and dry weight by maintaining leaf relative water content, as it was demonstrated in chrysanthemum flowers [12]. Putrescine also alleviates oxidative stress by reducing the levels of malondialdehyde and hydrogen peroxide and enhancing the activities of antioxidant enzymes such as ascorbate peroxidase [13]. By binding to negatively charged phospholipid head groups in membranes, putrescine enhances membrane stability and permeability-exerting dual roles critical for extending the longevity of cut flowers [14]. Last but not least, delaying senescence by putrescine further supports flower life after harvesting [15].

Senescence is a genetically regulated process that is manifested at the cellular or whole-plant level. These events are characterized by the increase in oxidative stress, cessation of photosynthesis, deterioration of organelle ultrastructure, and breakdown of some key cellular components including chlorophyll, proteins, lipids, and cell wall materials. These changes lead to loss in integrity of the cell membrane and loss in cellular and tissue structure [16]. The change from optimal growing conditions to postharvest conditions has a dynamical effect on the changes in quality of harvested products. Over the past two decades, however, there is an enormous improvement in postharvest physiology toward reducing losses and maintaining quality and promoting better handling, storage, transport, and distribution of these ornamental commodities. These developments notwithstanding, postharvest senescence remains one of the major factors that limit the marketability of cut flowers [17].

In light of their long history of being useful, this present work has chosen melatonin and putrescine as points on which to hinge in the quest for extending postharvest longevity and quality improvement in cut flowers. Being a naturally occurring antioxidant, melatonin was previously reported to delay senescence, both oxidative stress-induced and -mediated leaf, through membrane stabilization, enhancement of antioxidant defense, modulation of PPO activity among other enzymes. These mechanisms have been reported to extend vase life and reduce postharvest losses in numerous flower species [7]. Besides, non-toxicity and the effectiveness of melatonin as a means of minimizing the use of synthetic chemicals consider this agent an ideal candidate for sustainable postharvest treatments in the flower industry. Putrescine is a polyamine due to its several mechanisms that delay senescence. It stabilizes cellular membranes, reduces ethylene production, and improves water uptake-all factors considered very important in maintaining flower freshness [10,11]. The use of putrescine is also cost-effective and an environmentally friendly approach, hence offering a greener alternative to the usual chemical preservatives used within the floral industry. Both melatonin and putrescine have consequently been given recent attention as two promising, environmentally-friendly ways to achieve the goal of quality improvement in extending the vase life of fresh-cut flowers to meet the increasing demand associated with sustainable agriculture.

Alstroemeria is one of the most important cut flowers for its bright colors and beautiful appearance. However, rapid senescence and deterioration usually shorten the commercial life of this plant after harvest [18]. In this respect, vase life extension and quality maintenance during storage depend on maintaining physiological and biochemical properties important for cut flower longevity [19]. Growth regulators like melatonin and putrescine have been tried with promising responses in promoting postharvest longevity and tolerance to stress in many plant species [20,21]. However, there is scant literature regarding their individual applications on Alstroemeria flowers at varying concentrations. It is expected that the optimal concentrations of melatonin and putrescine treatments would improve physiological and biochemical stability, leading to extended vase life and aesthetic quality in Alstroemeria 'Amatista'. The main objective of the present investigation will be to observe the effect of exogenous melatonin and putrescine at varied concentrations on various physiological, biochemical, and postharvest aspects of said flowers. It attempts to search out the best treating conditions for longevity promotion, maintaining water relations, dimming oxidative stress, preserving membrane integrity in cut flowers with a postharvest management view.

## 2 Materials and methods

### 2.1 Plant material and growth conditions

**Fig. 1:**
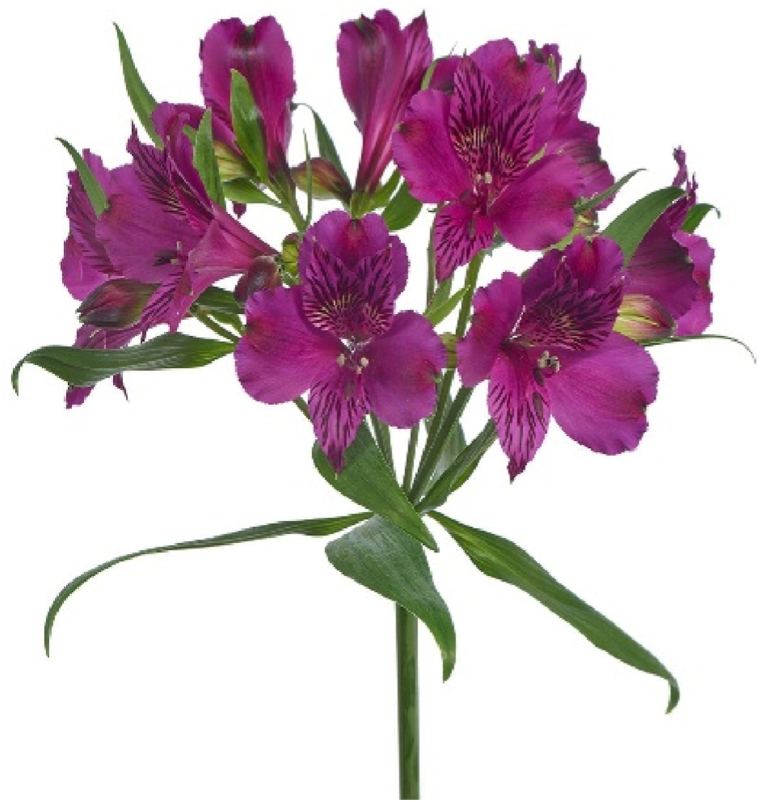
Alstroemeria ‘Amatista’ flower (https://www.royalvanzanten.com).

This experiment was conducted in the Department of Horticultural Science, Urmia University, using Alstroemeria flowers cv. Amatista (Royal Van Zanten, Netherlands) (Fig. 1). Rhizomes of Alstroemeria, purchased from a commercial greenhouse in Varamin, Iran, were planted in 24 cm diameter × 19 cm tall plastic pots in a soilless medium of perlite and cocopeat in a 1:3 volumetric ratio. Greenhouse conditions were maintained at a maximum/minimum temperature of 18–21 °C/10–13 °C, respectively. The light duration was 10–12 h with an intensity of 400–500 μmol m⁻^2^ s⁻^1^. Flowers were harvested during the early morning when one to two buds had opened. Immediately after harvest, the stems were brought to the laboratory, cut under running water to uniform lengths of 50 cm, and placed in 400-ml vases containing distilled water (control) or solutions of melatonin or putrescine with 4 % sucrose. Vase solutions were not changed throughout the experiment. The flowers were kept under environmental conditions of 22 ± 1 °C, relative humidity of 70 %, and a photoperiod of 12 h. The light intensity was 13 μmol m⁻^2^ s⁻^1^, supplied by fluorescent lamps. Assessments of the flowers were done on the first, 6th, and 12th day.

### 2.2 Experimental design

This was a factorial experiment under a completely randomized design, in which two factors were assessed: application of growth regulators as vase solutions, with five levels-0 (control), melatonin from Sigma-Aldrich at 100 and 200 μM, and putrescine from Sigma-Aldrich at 1.5 and 3 mM. The second factor was time of evaluation in the vase life period, having done evaluations on the 1st, 6th and 12th days. There were three replicates, with three observations each. Each experimental unit was made up of three vases, each with one flower, for a total of nine flowers per treatment. Concentrations of melatonin and putrescine were chosen in accordance with the data published previously for such type of experiments ([5,7]).

### 2.3 Physiological indices

#### 2.3.1 Electrolyte leakage (EL) of petals

For the determination of electrolyte leakage (EL), 0.2 g of petal tissue was washed in deionized water and incubated in 15 ml of deionized water at 40 °C for 30 min. The initial electrical conductivity (EC1) was read using a conductivity meter. Afterward, the samples were heated at 100 °C for 10 min, cooled, and the final conductivity (EC2) was measured. Electrolyte leakage was then calculated using formula [22]:

EL=(EC1/EC2)×100

#### 2.3.2 Relative water content (RWC) of petals

For the determination of RWC, petal discs (1 cm^2^ each) were weighed for fresh weight (FW) using an electronic balance with an accuracy of 0.0001 g. Then, they were floated on deionized water at 4 °C for 4 h, surface-dried, and weighed again to record the turgid weight (TW). Later, they were oven-dried at 72 °C for 48 h to measure the dry weight (DW). RWC was calculated by the following formula [23]:

$$RWC=\frac{FW-DW}{TW-DW}\times 100$$

#### 2.3.3 Relative fresh weight (RFW) of flowers

RFW was estimated weighing the flowers for the first time (FW₀) and then re-weighing at 6-day intervals (FWᵢ) until termination of vase life. RFW was calculated by the formula of Joyce and Jones [24] expressed as:

RFW=FWi/FW0

#### 2.3.4 Relative solution uptake (RSU) of flowers

The weight of flowers on day 1 with the volume of solution record at each observation gave an index of solution uptake for days 6 and 12. RSU was calculated as by Alaei et al. [25],:

$$RSU=\frac{WUi}{FW0}$$

RSU: Relative Solution Uptake, WUi: The absorbed solution on the specified day (ml), FW0: The initial weight on the first day (g).

### 2.4 Biochemical indices

#### 2.4.1 Total anthocyanin content of petals

For anthocyanin content determination, 0.1 g of petal discs were homogenized in 10 ml of acidic methanol (methanol containing 1 % HCl) and incubated in the dark at 25 °C for 24 h. The extract was centrifuged at 4000 rpm for 10 min, and the supernatant was measured for absorbance at 550 nm using a spectrophotometer (Dynamico, HALO DB-20). The anthocyanin concentration was then calculated using the extinction coefficient (ε = 33,000 cm^2^/mol) with the following formula and expressed in μmol/g fresh weight (FW) [26].

A=εbc

A: represents sample absorption, b: is the spectrophotometer's cell width, and c: denotes the concentration of the solution.

#### 2.4.2 Malondialdehyde (MDA) content of petals

Malondialdehyde content was determined by grinding 0.2 g of petal tissue in 5 ml of 1 % trichloroacetic acid (TCA). The homogenate was centrifuged at 8000 rpm for 10 min and 1 ml of the supernatant was mixed with 4 ml of 20 % TCA containing 0.5 % thiobarbituric acid (TBA). This mixture was then heated at 95 °C for 30 min, rapidly cooled on ice, and centrifuged again at 8000 rpm for 5 min. Absorbance was measured at 532 nm and 600 nm and MDA content calculated using the following formula [27]:

MDA(μmol/gFW)=(A532–A600/155)×100

#### 2.4.3 Hydrogen peroxide (H₂O₂) content of petals

**Fig. 2:**
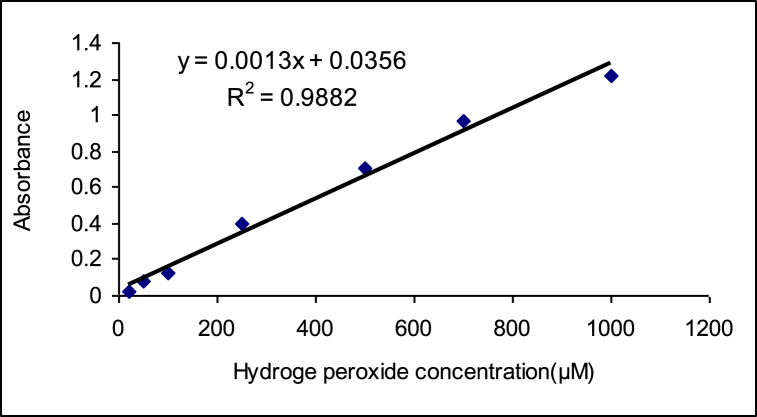
Standard curve of Hydrogen peroxide.

Hydrogen peroxide (H₂O₂) content was determined by the oxidation of potassium iodide by H₂O₂. Tissue of petals (0.5 g) was ground in 0.1 % trichloroacetic acid (TCA) and then centrifuged for 15 min at 12000 rpm. Besides, a reaction mixture consists of 0.5 ml of 10 mM potassium phosphate buffer pH 7.0, 1 ml of 1M KI and 0.5 ml of supernatant. The absorbance at 390 nm was measured and the concentration of H₂O₂ was estimated by using the standard curve obtained [28] (Fig. 2).

Y = 0.0013X+0.0356.

Y: The number read on the spectrophotometer.

X: The amount of hydrogen peroxide in μM/g F.W.

#### 2.4.4 Phenylalanine ammonia-lyase (PAL) activity of petals

Activity of phenylalanine ammonia-lyase was assayed by the method of Zucker [29]. Tissue of petals (0.5 g) was homogenized in 1.5 ml of buffer for extraction, containing 0.1 M borate buffer, pH 7.0, 0.1 % polyvinylpyrrolidone, and 1.4 mM 2-mercaptoethanol. The homogenate was centrifuged at 12,000 rpm for 15 min at 4 °C. The reaction mixture consisted of 30 μl of enzyme extract, 1 ml of 0.1 M borate buffer, pH 8.8, and 1 ml of 12 mM L-phenylalanine. Absorbance was read at 290 nm, and the activity of PAL was determined from the extinction coefficient (ε = 9630 μl/cm), expressed as nmol/min/g fresh weight (FW).

#### 2.4.5 Polyphenol oxidase (PPO) activity of petals

PPO activity was assayed as described by Kar and Durgesh [30]. Petal tissue (0.5 g) was homogenized in 0.15M Tris buffer, pH 7.5, which contained 50 mg polyvinylpyrrolidone. The homogenate was centrifuged at 14,000 g for 10 min at 4 °C. Aliquots (50 μl) containing 100 m M pyrogallol, were mixed with 3000 μl of phosphate buffer, pH 7, with 50 μl of enzyme extract. Absorbance was read at 420 nm, and the activity of PPO was calculated using the formula:

$$\text{PPO}\;A\text{ctivity}\;\left(U/\text{mg}\;\text{Protein}\right)=\frac{⧍A420\times Vt\times df}{\epsilon \times I\times t\times Vs}$$

U: Enzyme unit.

ΔA420: Change in absorbance at 420 nm (A420 after reaction minus A420 before reaction)

Vt: Reaction volume (in liters)

df: Dilution factor (if the sample was diluted)

ϵ: Molar extinction coefficient (the molar absorptivity coefficient, typically determined experimentally)

I: Path length of the light in the cuvette (usually in centimeters)

t: Reaction time (in minutes)

Vs: Sample volume (in liters)

### 2.5 Vase life of flowers

Vase life was defined as the period during which cut flowers retained their commercial and ornamental value, until either 50 % of the leaves turned yellow or 50 % of the florets dropped [31,32]. The flowers were observed daily using these parameters.

### 2.6 Statistical analysis and software utilized

The ANOVA and mean comparison for the studied traits were done by SAS software ver. 9.2. The factorial experiment was carried out in a completely randomized design with two factors, each with three replications and three observations for each replication. Multiple range tests were performed by using Tukey's test at a probability level of 1 %. In addition, the development of relationships of the traits was enhanced with heatmap analysis, principal component analysis, and Pearson correlation using the R Studio software.

## 3 Results and discussion

### 3.1 Ion leakage of petals

**Fig. 3:**
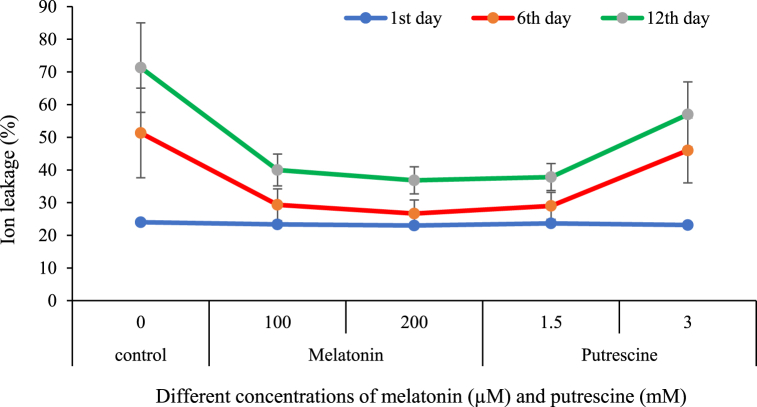
Effect of different concentrations of putrescine and melatonin during vase life period on ion leakage of petal in Alstroemeria 'Amatista'.

It was found that ion leakage increased with the increase of time in cut flowers of Alstroemeria. The putrescine and melatonin treatments were effective in reducing this increase compared to the control. Melatonin treatments at 100 and 200 μM, as well as putrescine treatment at 1.5 mM, effectively lowered the increase of ion leakage with time. In Fig. 3, the maximum ion leakage (71.33 %) was recorded in the control flowers on day 12.

The increase in ion leakage of cut flowers is mainly brought about by senescence-induced degradation of membranes. The phospholipid bilayer of the cell membrane regulates ion movement, and this integrity is lost during senescence because of mechanical injury, activation of degrading enzymes, and hormonal changes. This degradation is enhanced by phospholipases, and changes in the hormone levels, especially the build-up of ethylene, result in high membrane permeability, which manifests as increased ion leakage [33].

This study demonstrated that applying melatonin was able to drastically decrease ion leakage of cut Alstroemeria flowers by improving the integrity of their membranes. This protection in the current study is related to the possibility of melatonin to reduce levels of lipid peroxidation, oxidative stress, and acts by stabilizing a variety of cell structures [34]. The least ion leakage rate observed in 100- and 200-μM treatments agrees with previous reports pointing out that this molecule plays its role in an improvement of mitochondria function as well as an ability to scavenge ROS [35]. Results therefore have shown that melatonin could act as a natural elicitor for improving the quality and extending the vase life of cut flowers through its participation in membrane stability and mitigation of oxidative damage. These results are in agreement with previous studies by Lezoul et al. [7] in carnation, Wang et al. [35] in peony, Farooq et al. [10] in snapdragon, and SeyedHajizadeh et al. [36] in gerbera that reported the efficiency of melatonin for maintaining cellular integrity and delaying senescence in cut flowers.

Similarly, putrescine increases membrane stability through the interaction of free radicals with membrane phospholipids by free radical scavenging [37]. This polyamine inhibits degradation of membranes occurring during senescence, reducing ion leakage, thus allowing cell turgor [38]. Besides, under putrescine treatment, cellular pH is stabilized, together with ion homeostasis, this effect of putrescine [14]. Our results are in good agreement with those reported in the literature for a decrease in ion leakage, MDA accumulation, and oxidative damages along with enhancement of antioxidant enzyme activities in cut flowers treated with putrescine ([13,38]).

### 3.2 Relative water content (RWC) of petals

**Fig. 4:**
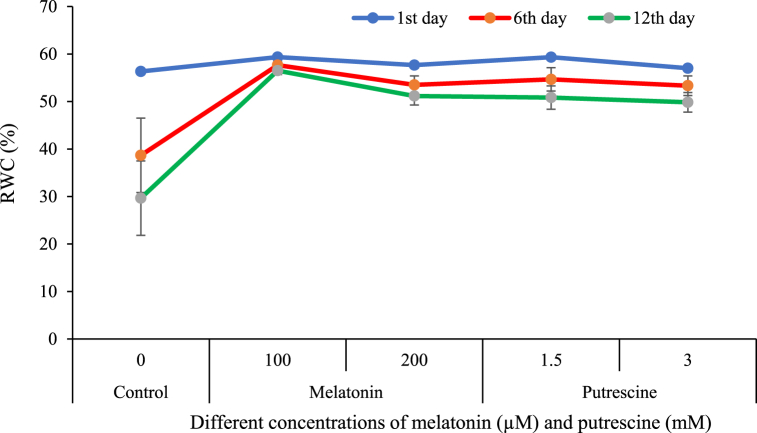
Effect of different concentrations of putrescine and melatonin during vase life period on RWC of petal in Alstroemeria 'Amatista'.

Fig. 4 illustrates that RWC of petals decreased during the time course, but in flowers pre-treated with either putrescine and melatonin, this decrease was not significant in the storage course. In the control flowers, however, there was a significant RWC reduction in comparison with the treated flowers. The lowest RWC (29.66 %) was recorded on the 12th day in the control, without any treatment by putrescine or melatonin (Fig. 4).

### 3.3 Relative fresh weight (RFW) of flowers

**Fig. 5:**
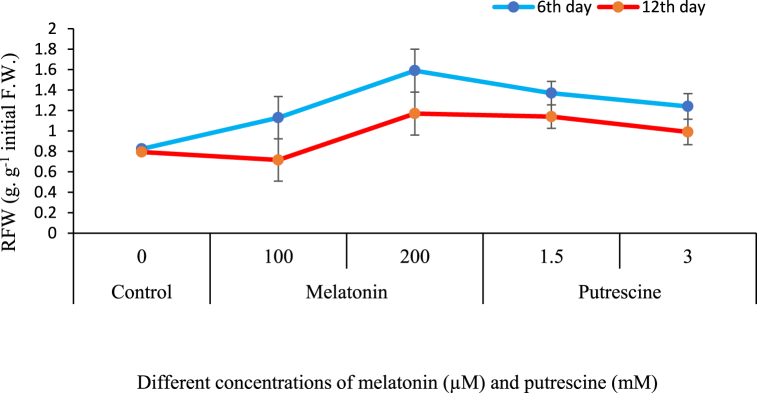
Effect of different concentrations of putrescine and melatonin during vase life period on RFW of flowers in Alstroemeria 'Amatista'.

The results of the present experiment represent the gradual decline of RFW during the vase life of cut flowers of Alstroemeria. However, all treatments resulted in higher RFW compared to the controls, and the highest effects were observed in 1.5 mM putrescine and 200 μM melatonin. The greatest effectiveness was found in the case of treatment with 200 μM melatonin on day six in reducing the loss of RFW. By day twelve, 200 μM melatonin and 1.5 mM putrescine were equally effective in maintaining RFW (Fig. 5).

### 3.4 Relative solution uptake (RSU) of flowers

**Fig. 6:**
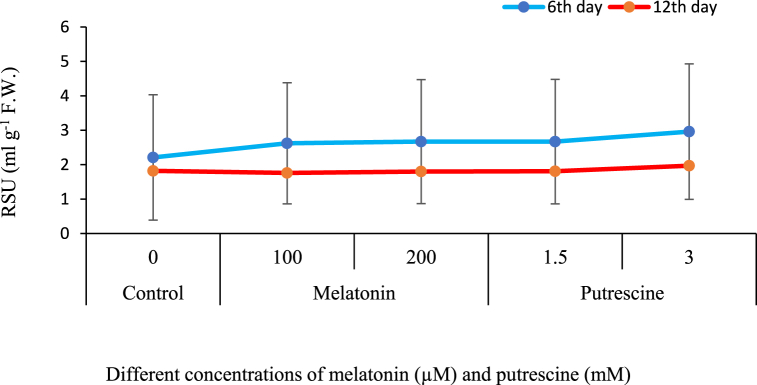
Effect of different concentrations of putrescine and melatonin during vase life period on RSU of flowers in Alstroemeria 'Amatista'.

Fig. 6 shows the temporal decline in relative solution uptake of cut Alstroemeria flowers. Nevertheless, putrescine and melatonin treatments significantly enhanced solution uptake compared to the control throughout the postharvest period. Among the treatments, 1.5 mM putrescine showed the most profound effect up to day six. On day twelve, all treatments showed similar effects on solution uptake (Fig. 6).

In the current study, RWC of petals, RFW of flowers, and relative solution uptake decreased in control plants due to loss of cell membrane integrity from various postharvest factors: increased ion leakage and water loss associated with degradation of cell membranes [39], and increased respiration rates in the petals, converting sugars into water and carbon dioxide, which accelerates the water loss process [40]. These processes are influenced by hormonal imbalance, especially of ethylene and auxin, which increase respiration and ion leakage [41]. Blocking of xylem vessels in the flower stems also impedes solution uptake, limiting water delivery to the petals [33]. All these factors result in a considerable decline in both RWC and RFW, and water stress is central to this process [42].

Both melatonin and putrescine enhanced these losses by sustaining petal RWC and maintaining RFW and solution intake. Melatonin treatment induced these responses via the induction of ion leakage reduction, hormonal balance, inhibition of ROS production, improvement of water intake, and stimulation of protein synthesis [43]. These mechanisms not only prevented weight loss and maintained the freshness of the stems but also improved water uptake, as manifested in their extended vase life [44]. These findings agree with the reports of melatonin reducing water loss and improving postharvest longevity in ornamental plants ([5,7,35]). Putrescine also preserved RWC through the inhibition of ethylene production, maintenance of hormonal balance, prevention of cell membrane degradation, and enhancement of water uptake [12]. Besides, polyamines, of which putrescine is a part, inhibit microbial growth, prevent blockage of xylem, and enhance osmotic pressure within the petals; all these factors together prevent loss of weight and thus maintain the quality of flowers [14]. These activities result in reduced evaporation and respiration, hence preventing wilting and maintaining flower freshness [45]. Evidence from previous works supports that putrescine has indeed been proven to be able to maintain postharvest water relations, such as enhancing solution uptake and RWF in several cut flowers. For example, Kamiab reported that polyamines reduced water loss and increased solution uptake in carnations [46], while Ataei et al. [38] and Sedaghathoor et al. [15] observed similar effects of putrescine in preserving RFW and enhancing solution uptake in other ornamental species.

#### 3.4.1 Anthocyanin content of petals

**Fig. 7:**
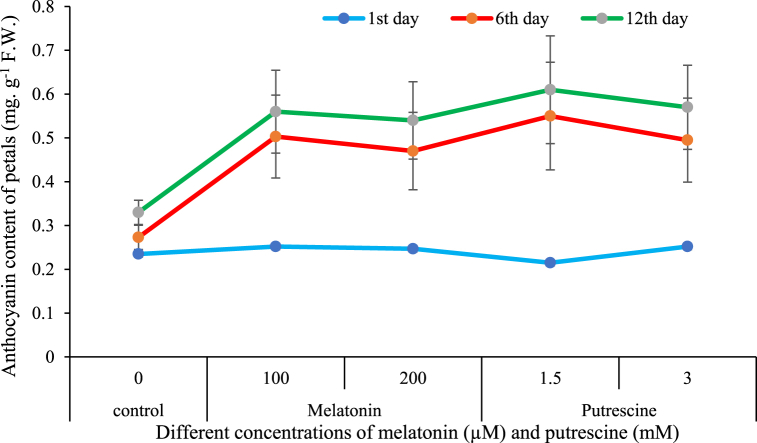
Effect of different concentrations of putrescine and melatonin during vase life period on anthocyanin content of petals in Alstroemeria 'Amatista'.

Fig. 7 illustrates that anthocyanin content of Alstroemeria flowers increased during the vase life period. Anthocyanin content was significantly higher in both putrescine and melatonin-treated flowers compared with the control. Maximum anthocyanin content was recorded in 100 μM melatonin and 1.5 mM putrescine-treated flowers.

Flower color is one of the most apparent features of cut flowers and is mainly hinged on plant pigments, which majorly include flavonoids, carotenoids, betalains, and chlorophylls. Of these, anthocyanins of the flavonoid subclass are the most abundant in some higher plant species and are remarkably responsible for coloration in many of those plant species [47]. However, changes in pigments during the postharvest period-inclusively anthocyanins-can alter the color of petals, depending on the species, and reduce their ornamental value. The dynamics of the pigments during the postharvest period are influenced by temperature, light, nutrition, and the composition of vase solutions, which include additives like sugars, salts, or plant growth regulators [48].

As shown in Fig. 7, extension of vase life of cut flowers of Alstroemeria was associated with an increase in anthocyanin content of the petals. This is promoted through several mechanisms: the stress imposed by harvesting induces synthesis of anthocyanins as a protective agent, protecting cells from damage. While this is occurring, degradation of chlorophyll during postharvest results in an increase in cytoplasmic pH, thus inducing pathways responsible for anthocyanin biosynthesis. Consistent with these observations, Lopez-Guerrero et al. described a reduction of chlorophyll content with the increase in anthocyanin accumulation in leaves of vase-treated lisianthus plants, which was in accordance with our findings regarding cut flowers of Alstroemeria [49]. The biosynthesis of anthocyanin is under hormonal regulation: ethylene promotes anthocyanin biosynthesis while abscisic acid can downregulate this process, decreasing the expression of biosynthetic genes. The light intensity also plays an important role in the accumulation: higher the intensity, the more intense the anthocyanin accumulation. Moreover, there is genetic variation among cultivars for the degree of anthocyanin production, where some cultivars are intrinsically able to synthesize more anthocyanins than others under postharvest conditions [50].

In this work, melatonin and putrescine treatments enhanced the anthocyanin contents in Alstroemeria petals. Melatonin upregulates genes that participate in anthocyanin biosynthesis and inhibits enzymatic degradation of anthocyanins, thus helping to keep the levels of anthocyanin in petals [51]. Besides, melatonin extends flower lifespan since it reduces oxidative stress and preserves cellular integrity, which together support flower color and quality [52]. These findings are in agreement with previous reports of the melatonin treatment on other cut flowers, including rose [5] and gerbera [36], where the anthocyanin content in cut flower petals increased during the vase life. Similar to this, polyamines induce anthocyanin biosynthesis and enhance pigment retention in cut flowers. Indeed, evidence is available that proves polyamines elevate anthocyanin levels and overall pigment concentration during the postharvest period. These findings indicate that polyamines have the potential to improve the aesthetic and commercial value of cut flowers by maintaining intense petal coloration and extending vase life ([14,53]).

#### 3.4.2 Malondialdehyde content of petals

**Fig. 8:**
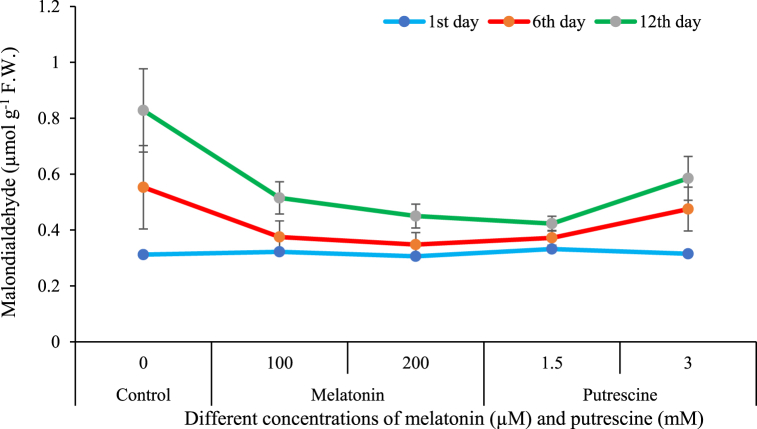
Effect of different concentrations of putrescine and melatonin during vase life period on malondialdehyde content of petals in Alstroemeria 'Amatista'.

The mean comparison shows that the increased vase life of flowers in the preservative solution has increased the accumulation of MDA in the petals. On the other hand, putrescine and melatonin treatments prevented this increase in comparison with the control. Putrescine at 1.5 mM and melatonin at 200 μM were effective treatments that prevented the rise in MDA levels in the treatments. Especially, there was a significant reduction in the accumulation of MDA, being almost 50 %, for those subjected to treatment with 1.5 mM of putrescine as opposed to that found in the control (Fig. 8).

In the present study, postharvest maintenance of Alstroemeria cut flowers in vase solutions enhanced MDA, a critical indicator of oxidative breakdown of lipids in plant cells. Detachment of cut flowers acts as a stress factor for induction of physiological response in the form of accumulation of ROS, accelerating senescence due to cellular damage. Excessive production of ROS disrupts the balance between generation and scavenging of ROS, leading to peroxidation of lipids and MDA formation [54]. The stress-induced declines in the activities of antioxidant enzymes further promote oxidative damage, and degradation of membranes releases MDA into the extracellular environment. The rise in MDA levels across the postharvest period in the flowers of the untreated control confirms such established stress responses [55].

Senescence is an irreversible genetic process which takes place at the cell, tissue, organ, and whole-plant levels. It involves oxidative disorders such as inhibition of photosynthesis, degradation of organelles, loss of chlorophyll and proteins, increase in oxidation of lipids, decomposition of cell wall components, and loss of membrane integrity. All these changes together provide structural and functional decline in cells and tissues.

Results in this study indicated that melatonin treatment significantly alleviated cell membrane lipid peroxidation. Previous studies have illustrated that exogenous melatonin components increase the activity of antioxidant enzymes and decrease ROS, such as hydrogen peroxide and superoxide radicals. It regulates ROS metabolic balance, which in turn reduces lipid peroxidation by maintaining low levels of MDA and preserving membrane integrity. For example, exogenous melatonin treatments reduce the MDA levels in lisianthus ([38,56]), tuberose [6], and peony [35]. Ma et al. [57] also reported cell membrane stability and a reduction in oxidative damage by reducing the MDA concentration. Its role in neutralization of ROS and prevention of oxidation of unsaturated lipids results in a decrease in the oxidative stress status and increased resistance of lipids to peroxidation. This mechanism could account for the reduced ion leakage and MDA levels in our study. ROS scavenging and prevention by melatonin keep cell membranes from oxidative degradations caused by them too [58].

Flowers treated with 1.5 mM putrescine showed considerably lower ion leakage and MDA content than controls, indicating that putrescine reduces the peroxidation of lipids and protects cellular structures. As polyamines, including putrescine, are anti-senescence in nature, by virtue of their inhibitory action on biosynthesis of ethylene and acting like cytokinins, they prolong longevity in plants. At the molecular level, polyamines inhibit cell membrane disruption by reducing ROS levels through their antioxidant action and inhibiting enzymes such as lipoxygenase responsible for the breakdown of membrane lipids ([10,59,60]). This inhibition prevents the loss of membrane permeability and enhances quality in flowers [55]. Secondly, polyamines inhibit the formation of ACC oxidase responsible for converting ACC to ethylene, a restrictive process that leads to the decrease of free radicals across cell systems [61]. These mechanisms underpin the role of polyamines in reducing oxidative stress and delaying senescence by preserving cellular and membrane integrity. Supporting these findings, other basic research on other cut flowers like gerbera [13], lisianthus [38], and the cut foliage of sword fern [62] showed that exogenous polyamine reduced MDA content in petals.

#### 3.4.3 The level of hydrogen peroxide (H_2_O_2_) of petals

**Fig. 9:**
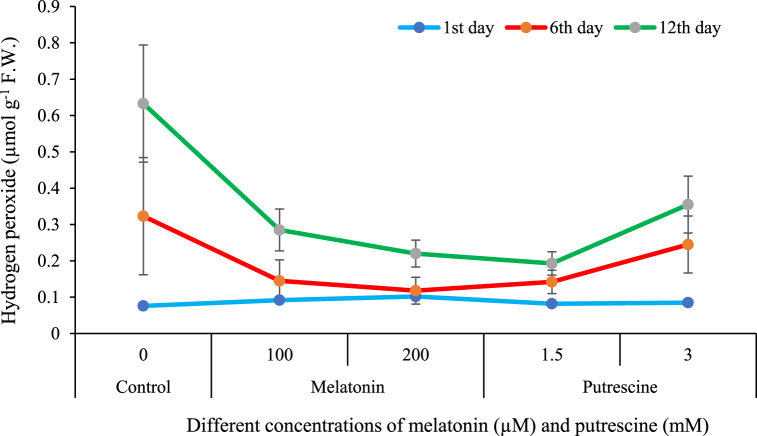
Effect of different concentrations of putrescine and melatonin during vase life period on hydrogen peroxide of petals in Alstroemeria 'Amatista'.

Fig. 9 illustrates that hydrogen peroxide levels increased overtime in both control and treated Alstroemeria flowers. However, treatments containing either putrescine and melatonin delayed this increase. More precisely, among the treatments studied, 100 and 200 μM melatonin treatments, and 1.5 mM putrescine treatment were much more effective in decreasing hydrogen peroxide production on the sixth and twelfth days, respectively. The highest level of hydrogen peroxide, 0.633 μM g⁻^1^ F W., was recorded in the control flowers on the twelfth day. However, the application of 1.5 mM putrescine reduced the levels of hydrogen peroxide by about 3.5 folds compared to the control.

Hydrogen peroxide (H_2_O_2_) is one of the most important molecules in plant physiology: at low levels, it may serve as a signaling molecule, while higher concentrations are highly damaging [63]. While it regulates gene expression, growth, and stress responses under mild oxidative stress conditions, its accumulation induces oxidative damage to proteins, lipids, and DNA, thus accelerating cell death and senescence. In cut flowers, excessive build-up of H_2_O_2_ has been directly linked to wilting and reduction of vase life [64]. The current study revealed that older aged flowers continuously showed enhanced levels of H_2_O_2_; this could be favored by enhanced activity of NADPH oxidases, leakage of lipoxygenase enzymes due to degradation of membranes, and reduced activities of H_2_O_2_-degrading enzymes like catalase and peroxidase under postharvest stress conditions [65,66].

Bioactive roles of melatonin and putrescine vase solution treatments are involved in both enzymatic and nonenzymatic alleviation of H_2_O_2_ within the cut flowers of this present work. Melatonin treatment enhances the activity of key antioxidants, catalase and peroxidase, which have a major role in the dissection of H_2_O_2_ during attenuation by melatonin in oxidative damage [44]. The production of H_2_O_2_ is inhibited because melatonin represses those enzymes responsible: NADPH oxidases and lipoxygenases. Our group has reported that melatonin delays senescence, modulates ABA biosynthesis, an important hormone with a close relationship to the senescence of petals, and upregulates antioxidant pathway genes [67]. Such effects have been used to explain how melatonin works in prolonging the vase life in tuberose flowers [6], rose flowers [5], and carnation flowers [7].

Similarly, putrescine mitigates the levels of H_2_O_2_ through upregulating the expression of antioxidant genes involved in CAT and POD activities while inhibiting NADPH oxidase and lipoxygenase activities. The hormonal regulating role played by putrescine mainly through its reducing effects on ethylene and ABA levels maintain cell integrity and hence diminished oxidative stress [68]. Putrescine also exerts its protective effect on membranes, avoiding degradation and inducing metabolic pathways such as γ-aminobutyric acid (GABA) shunt involved in H_2_O_2_ detoxification [69,70]. Indeed, applications of putrescine significantly delayed wilting in sword fern (*Nephrolepis cordifolia*) [62] and lisianthus [38] by decreasing H_2_O_2_ levels.

In other words, melatonin and putrescine promote antioxidant defense systems, repress the production of H_2_O_2_-producing enzymes, and modulate stress-related hormones in a way that together decreases oxidative damage and increases the vase life of cut flowers. Even though melatonin has higher antioxidant capacity, the actions of putrescine are complementary to those of melatonin via different pathways, hence their potential in postharvest treatments.

#### 3.4.4 Phenylalanine ammonia-lyase (PAL) enzyme activity of petals

**Fig. 10:**
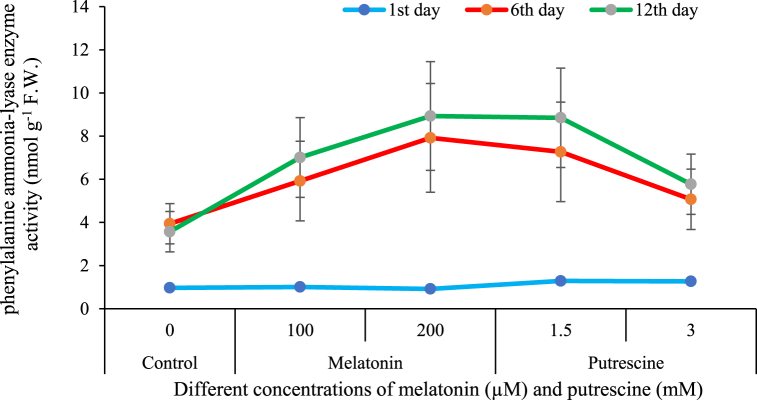
Effect of different concentrations of putrescine and melatonin during vase life period on phenylalanine ammonia-lyase enzyme activity of petals in Alstroemeria 'Amatista'.

Results are presented to show that, within this period, the level of activity of the enzyme PAL was enhanced, especially with time and against the treatment with putrescine-melatonin treatment. On day one, generally, the effect of PAL on the flowers remains the same across all flowers. However, the treatments varied in flower longevities. Fig. 10 shows that both melatonin concentrations and 1.5 mM putrescine treatment have the same effects, increasing PAL enzyme activity on the sixth and twelfth days, significantly enhancing PAL activity as compared to the control.

PAL is a key enzyme in the phenylpropanoid pathway, catalyzing the conversion of phenylalanine to trans-cinnamic acid, which is a precursor for flavonoids, lignins, and anthocyanins [71]. PAL has been widely reported to take part in postharvest flower longevity mainly due to its implication in ROS and regulation of stress-related compounds. The activity of PAL increases during early senescence, which is part of the flower defense mechanism, but in later stages of senescence, PAL activity decreases due to cellular breakdown and reduced enzyme synthesis, which results in quality deterioration such as wilting and edge browning [72].

In the present work, melatonin and 1.5 mM putrescine treatments enhanced PAL activity in cut flowers. Melatonin enhanced the expression of genes of PAL that in turn promote higher synthesis and activity of the enzyme [7]. In amaryllis [73], gerbera [36] and carnation [7] flowers it was reported that melatonin treatment-maintained PAL activity under stress conditions and improved post-harvest quality. Similarly, putrescine enhanced the activity of PAL by mitigating oxidative stress, regulating stress hormones, and increasing the expression of PAL genes [74]. In gerbera, too, enhanced activity of PAL upon exogenous polyamine application has been suggested to occur under increased levels of the enzyme [75]. However, at higher concentrations-for example, 3 mM-putrescine acted oppositely, suppressing PAL activity, which may be toxic to flower cells, as evident from our studies.

### 3.5 Polyphenol oxidase (PPO) enzyme activity of petals

**Fig. 11:**
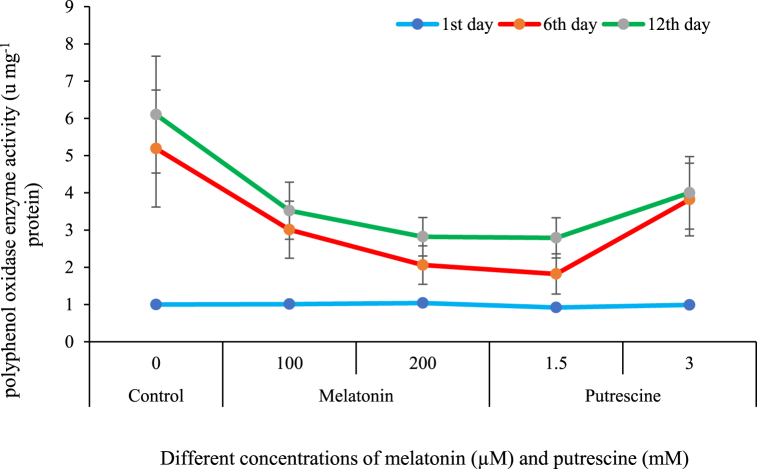
Effect of different concentrations of putrescine and melatonin during vase life period on polyphenol oxidase enzyme activity of petals in Alstroemeria 'Amatista'.

Regarding the data comparing the averages, it is shown that in a chain of events relating to senescence in Alstroemeria flowers (cut), one constant uprising respect to polyphenol oxidase was higher from one time interval of test initiation to the successive one, thus evidencing increases up through each stage and across time frames considered. Such changes over time were hindered by applications of putrescine and melatonin compared to a control without treated applications. In particular, 1.5 mM putrescine and 100 and 200 μM melatonin concentrations were more effective in preventing the increase of PPO enzyme activity. According to Fig. 11, the highest values of PPO activity were obtained from the control flowers on both the sixth and twelfth days.

Polyphenol oxidase is considered one of the major plant enzymes responsible for the oxidation of phenolic compounds, which generally results in the browning and/or reduction of vase life. Major ways PPO acts in the postharvest deterioration of flowers include a) Browning of flowers accelerates senescence due to cell membrane injury leading to cell death and thus shortening flower longevity. b) Color changes: Browning of the edges of the petals, very characteristic in light-colored flowers. (c) Loss of visual quality, rendering flowers less marketable [76]. Among the various factors that increase PPO activity in cut flowers are mechanical damage such as cutting of stems, oxidative stress, hormonal imbalance, including increased ethylene, and senescence-related gene expression upregulation of PPO. These changes enhance browning and deteriorate the general quality of flowers [77].

Melatonin and putrescine were reported to reduce PPO activity in cut flowers by reducing oxidative stress and modulating plant hormones, particularly ethylene. Melatonin reduces PPO activity by enhancing the antioxidant defense system and suppressing browning triggered by ethylene. In the case of putrescine, reduction in the levels of PPO is done by the action of antioxidant properties, modulation of gene expression coding for PPO, and modulation of ethylene, which together enhance the quality of flowers [78]. Results from this study confirm other studies showing that melatonin and polyamines, including putrescine, suppress PPO activities and enhance phenolic stability and antioxidant enzyme activities [14]. As an example, exogenous melatonin treatment to anthurium [79] and carnation cut flowers [7] reduced PPO activities during the vase life of flowers. Similarly, exogenous application of spermine at 2 mM has been reported to enhance phenol content and antioxidant enzyme activities like peroxidases and superoxide dismutases but reduce PPO activity, further supporting their role in maintaining postharvest flower quality [80].

### 3.6 Vase life of flowers

**Fig. 12:**
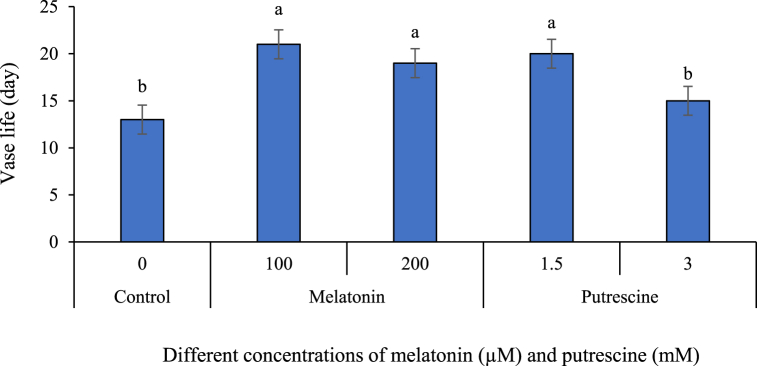
Effect of different concentrations of putrescine and melatonin on vase life of Alstroemeria 'Amatista' cut flowers. Different letters indicate significant differences at the 1 % probability level based on Tukey's test.

**Fig. 13:**
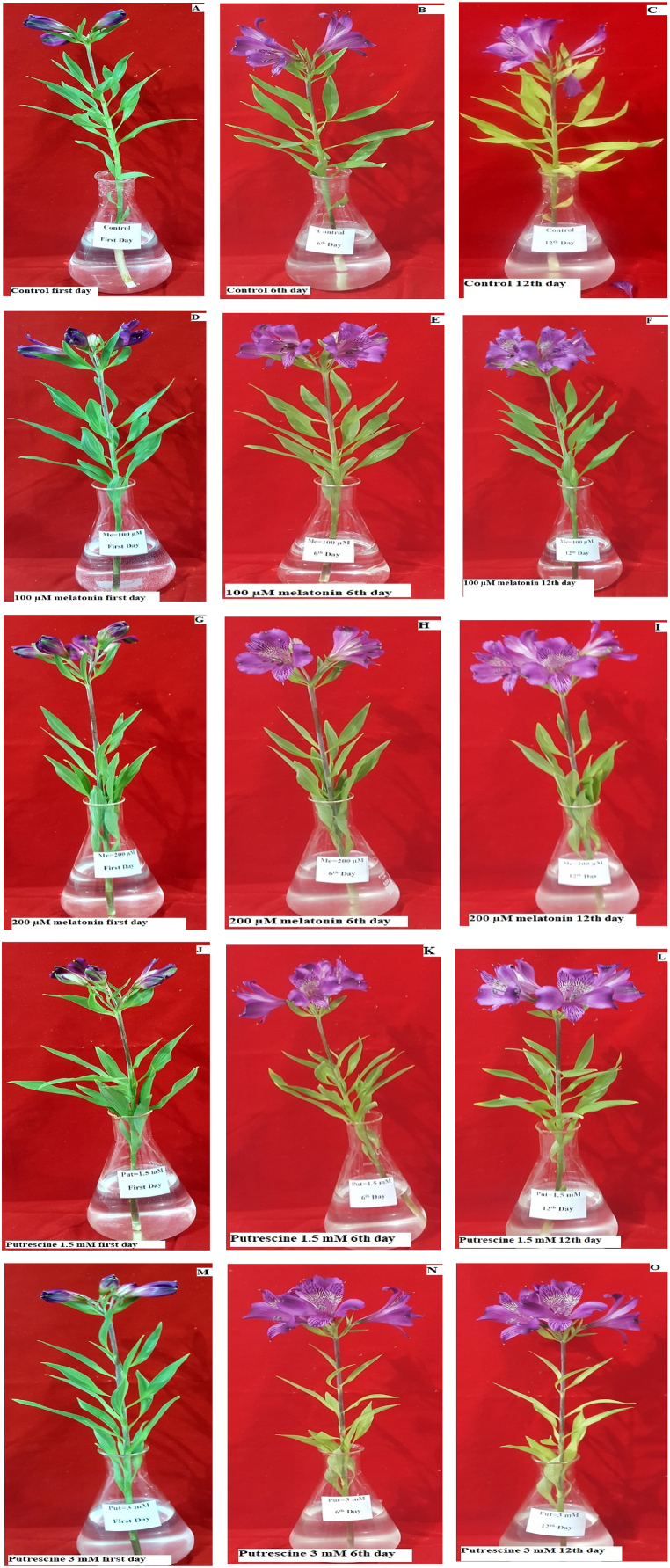
Effects of melatonin (100 and 200 μM) and putrescine (1.5 and 3 mM) on the vase life of Alstroemeria ‘Amatista’ flowers. Images (A–O) represent flower appearance across three time points: First day, 6th day and 12th day. (**A, B, C**: Control group (untreated) on First day, 6th day, and 12th day, respectively. **D, E, F**: Flowers treated with melatonin (100 μM) on First day, 6th day, and 12th day, respectively. **G, H, I**: Flowers treated with melatonin (200 μM) on First day, 6th day, and 12th day, respectively. **J, K, L**: Flowers treated with putrescine (1.5 mM) on First day, 6th day, and 12th day, respectively. **M, N, O**: Flowers treated with putrescine (3 mM) on First day, 6th day, and 12th day, respectively). The scale is 1:16, indicating that 1 cm in the image corresponds to 16 cm in reality.

In the work presented here, exogenous putrescine and melatonin in different concentrations were used to compare their effects on extending vase life in cut flowers of Alstroemeria 'Amatista'. The results obtained have shown that treatments with melatonin and putrescine significantly extended the vase life of cut flowers when compared to controls. In more detail, both 100 and 200 μM melatonin and 1.5 mM putrescine caused significant extension of vase life. In contrast, vase life of Alstroemeria was not affected by 3 mM application of putrescine (Fig. 12, Fig. 13).

Fresh-cut flowers are one of the most valuable crops in horticulture, and marketability relies on maintaining quality and extending vase life [81]. Vase life is determined by two major factors: ethylene, which promotes senescence [82,83], and impaired water uptake due to blockage in the stem, causing wilting [34].

Melatonin is a very powerful antioxidant, able to pass through cell membranes with ease [84], and was found to be very effective in maintaining the vase life of cut flowers. It has also been mentioned as one of the most powerful antioxidant molecules among growth regulators [85]. In this paper, melatonin treatments with 100 and 200 μM were found to greatly improve the vase life of Alstroemeria cut flowers. This is attributed to mitigation of oxidative stress caused by melatonin, stabilization of cell membrane, maintenance of water balance, and activation of the antioxidant enzyme. It is proposed that the basis for this activity may be explained by a reduced PPO activity, although it is not exactly known how melatonin interacts with PPO, which could contribute to explaining the role of melatonin in the extension of the vase life of cut flowers. These are in agreement with the studies of Mazrou et al. [5] in roses, Lezoul et al. [7] in carnations, Wang et al. [35] in peony, Seyedhajizadeh et al. [36] in gerbera, Zhou et al. [43] in carnations, Zainalipour et al. [56] in lisianthus, and Brito et al. [73] in amaryllis, on the positive effect of melatonin vase solution on maintaining cut flowers.

Polyamines including putrescine extend flower longevity by inhibition of the activity of ACC synthase, which prevents ethylene synthesis and maintains membrane stability [86,87]. Prevention of lipid oxidation and increase in the availability of sugars, proteins, and RNA in floral tissues are the reasons for the increased longevity granted by the polyamines to flowers [17]. Application of spermidine exogenously has been reported to delay senescence in cut carnation flowers significantly [53]. Similarly, Sardoei et al. [88] have reported that 100 mg/L putrescine significantly increased the vase life of narcissus flowers. Besides, Sedaghathoor et al. [15] also showed that the vase life of *Dianthus caryophyllus* cut flowers was significantly increased by applying putrescine. Haq et al. [17] reported that spermidine at 6 mM in vase solution significantly improved the vase life of gladiolus flowers compared to controls, which agrees with the findings of the present study.

A variety of factors before and after harvest influence the vase life of cut flowers. Better defense mechanisms, like improved solution uptake and maintenance of weight post-harvest, are important. Herein, 1.5 mM putrescine and 100–200 μM melatonin treatments enhanced the phenolic content and relative water content. While doing this, markers of senescence such as PPO activity, ion leakage, hydrogen peroxide levels, and malondialdehyde content were significantly reduced. Thus, these treatments extended vase life in Alstroemeria flowers from 12 to 13 days in control to 20–21 days. At a higher concentration of 3 mM, putrescine exhibited negative effects on the vase life of cut flowers, presumably due to its contribution to the enzymatic activity of PAL and PPO and other physiological changes. Higher concentration inhibited the activity of PAL, an enzyme involved in the phenylpropanoid pathway for the synthesis of critical plant compounds, thus weakening the flower's defense mechanisms and advancing senescence. This decrease in PAL activity resulted in reduced production of phenolics, which was crucial for maintaining flower quality and prolonging its vase life. Besides, an increase in PPO activities at this level promoted oxidative stress, causing greater membrane injury, browning of flowers, which was reflected negatively in the appearance of the flowers. PPO activities increased substantially, probably triggering a higher amount of MDA content-a product of lipid peroxidation reflecting more serious oxidative damage. Further, 3 mM putrescine enhanced ion leakage-a symptom of loss of cell membrane integrity-and reduced water uptake, with direct involvement in wilting development and consequently shortening the vase life. These combined effects of reduced antioxidant capacity, increased oxidative stress, and impaired water relations therefore suggest that the higher concentration like 3 mM putrescine is not effective in prolonging the vase life but instead accelerates the aging processes in cut flowers.

The results from this study present melatonin and putrescine as prospective environmental-friendly approaches for the extension of vase life in Alstroemeria cut flowers due to being one of the most important problems that affect the floriculture industry. These treatments improve physiological traits such as membrane stability, water relations, and the mitigation of oxidative stress apart from enhancing biochemical parameters like anthocyanin content and reducing markers of oxidative damage that are very important for maintaining postharvest quality. From a practical point of view, the scalability of these treatments assures promising opportunities from a commercial viewpoint. For instance, extending vase life from 12–13 days to 20–21 days with low concentrations of melatonin at 100–200 μM and putrescine at 1.5 mM would result in huge savings in postharvest losses, besides adding to marketability. However, there are also various challenges regarding large-scale adoption of treatments. While melatonin and putrescine are relatively inexpensive in laboratory use, more studies need to be done on the economic feasibility of these plant regulators at the commercial level with respect to the cost of production, formulation, and application logistics. Their wide application should also be studied in respect to their environmental impact and sustainability. Moreover, the optimization of protocols of these treatments according to environmental conditions, especially temperature and humidity, during transport and storage, is very important for enhancing their effectiveness at each level of the supply chain. Generally, the study points out the embedding of new, environmentally friendly treatments like melatonin and putrescine into user-friendly applications in floriculture. It will help the industry improve flower quality and consumer satisfaction while responding to the challenges of sustainability. Further studies may be directed at the complete explanation of the physiological and biochemical mechanism, evaluate the possibility of combining the treatment with other technologies-such as controlled atmosphere storage or biostimulants-for even greater improvements in postharvest conditions.

### 3.7 Heatmap clustering, pearson correlation, and principal component analysis

**Fig. 14:**
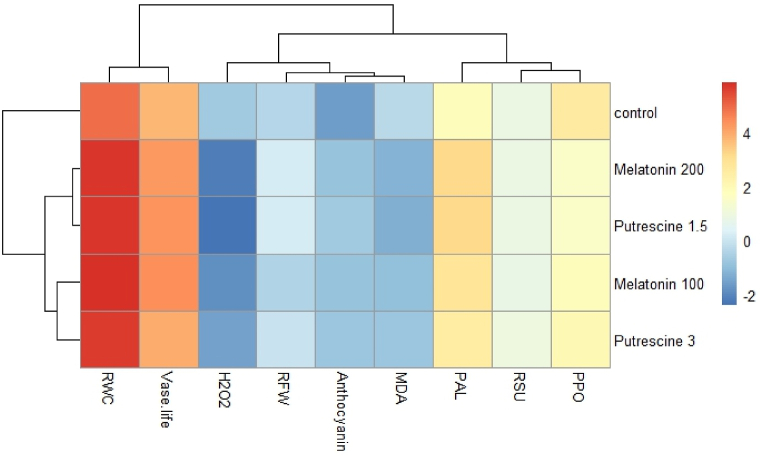
Heatmap clustering of growth regulators [Melatonin and Putrescine] on physiological and biochemical characteristics of cut flowers of Alstroemeria 'Amatista'. Color key: standardized mean values, dark red represents relatively high mean values, and dark blue represents relatively low mean values.

The heatmap illustrates the effects of melatonin treatments at 100 and 200 μM, putrescine at 1.5 and 3 mM each, as compared to the control on different postharvest physiological and biochemical indices taken from Alstroemeria 'Amatista'. In most instances, it so happens that lower indices-for example, RWC and vase life of control plants-are colored in light, hence suggesting limited hydration and longevity. On the other hand, 200 μM melatonin and 1.5 mM putrescine remarkably increased RWC and vase life, manifested by deeper red color tone, which indicates the enhancement of postharvest quality and stress tolerance. These treatments also lowered the contents of hydrogen peroxide (H_2_O_2_) and malondialdehyde (MDA), represented in blue color, indicating the low oxidative stress. Melatonin applied at 100 μM and putrescine at 3 mM had moderate effects, with partial strengthening of antioxidant enzyme activities (PAL, PPO), while having minor influences on oxidative damage markers. Overall, the clustering suggests that melatonin and putrescine treatments are superior to the untreated control in many aspects of postharvest quality, mainly at those concentrations which optimize hydration and reduce oxidative damage (Fig. 14).

**Fig. 15:**
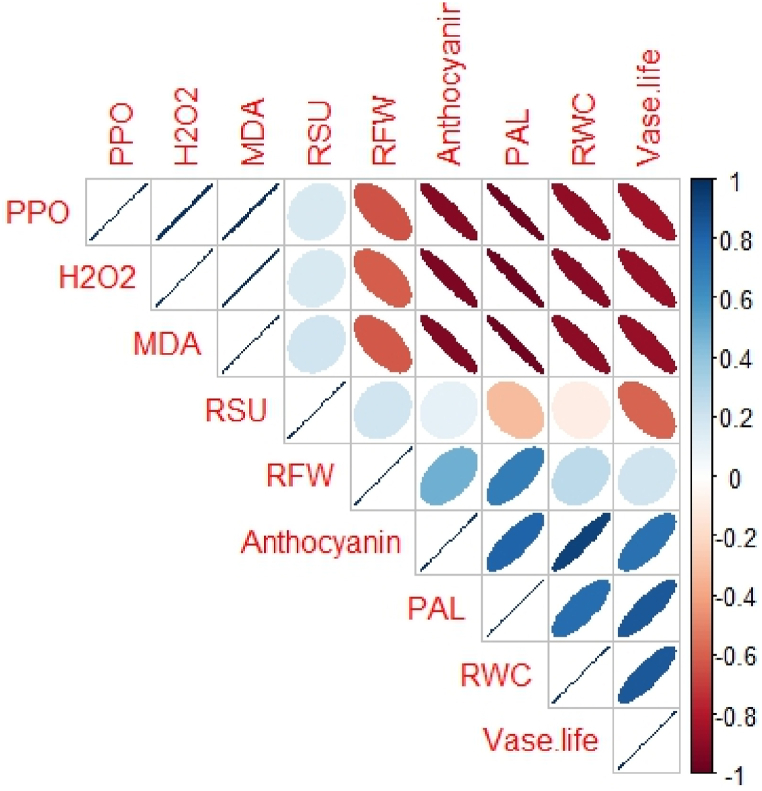
Pearson correlation heatmap describing correlations among several physiological and biochemical characteristics during postharvest stages of cut Alstroemeria 'Amatiata' flowers. The positive correlations are described by the blue ellipses, and the negative ones are depicted by the red ellipses. Color intensity and ellipse form also indicate how strong the correlations are; stronger correlations show more intensive color and smaller ellipses with a correlation coefficient from −1 to +1.

The correlation matrix describes the association between all the physiological and biochemical indices involved in postharvest Alstroemeria 'Amatista'. Vase life is highly positively correlated with RWC, PAL, and relative solution uptake, indicating that the higher hydration and enzymatic activities are the causes of the increased longevity of the flowers. On the other hand, vase life and RWC are negatively related to hydrogen peroxide (H_2_O_2_) and malondialdehyde (MDA), indicating that lower oxidative stress is associated with longer vase life. In addition, anthocyanin content is positively related to relative fresh weight (RFW) and PAL activity, which may indicate a protective role in maintaining flower quality. Generally speaking, improved hydration and reduced oxidative stress appear to be the most important factors for extending vase life in Alstroemeria (Fig. 15).

**Fig. 16:**
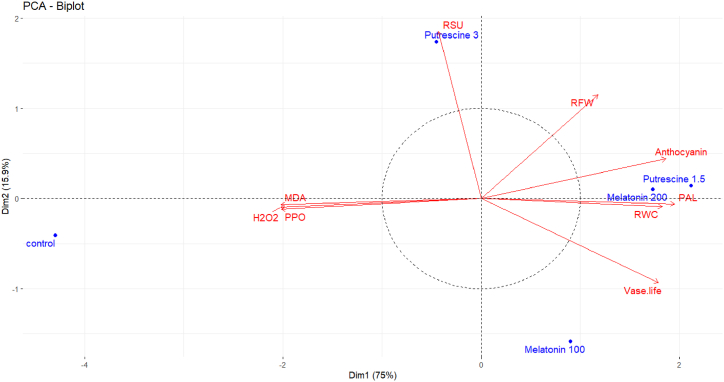
Principal Component Analysis (PCA) biplot of the distribution of physiological and biochemical characteristics of cut flowers Alstroemeria 'Amatista' submitted to melatonin and putrescine treatments in the postharvest stage. The plot shows the contribution of each characteristic to the principal components, underlining the relationships and variance among the characteristics. Each point represents a sample while the arrows indicate the direction and strength of the correlations between the characteristics. The traits that are closer to each other show stronger relationships, while traits that are farther apart suggest weaker correlations.

This PCA biplot displays the treatments of the melatonin and putrescine on postharvest characteristics of Alstroemeria 'Amatista'. The first dimension explains 75 %, while the second dimension explains 15.9 % of the total variance, reflecting a good separation between control plants and treated ones. Control pointed out in a separated position and showed the minimum value for almost all parameters. Putrescine at 3 mM has a very high association with RFW, anthocyanin, PAL activity, and RSU, which indicates its positive effect on the said characteristics. Melatonin at 200 μM and Putrescine at 1.5 mM were highly significantly associated with vase life and RWC of cut flowers, which means hydration and longevity improve significantly. In contrast, the oxidative stress markers H_2_O_2_ and MDA are negatively related to quality traits, emphasizing the treatments' function in reducing stress. Overall, the biplot highlights the efficacy of specific putrescine and melatonin concentrations in enhancing major postharvest characteristics of the commodity (Fig. 16).

## 4 Conclusion

The research work deeply studied the effects of melatonin and putrescine on vase life in Alstroemeria cut flowers. It was quite explicit from the results that both melatonin (at 100 and 200 μM) and putrescine at 1.5 mM significantly extended vase life in Alstroemeria as compared to the control. This was manifested by the prolongation of vase life from about 12 to 13 days in the control to 20–21 days in the treatments containing melatonin and/or putrescine. Surprisingly, the higher concentration of putrescine, 3 mM, had no significant effect on vase life. The underlying mechanisms for the extended vase life were quite varied, including physiological and biochemical processes. Melatonin easily crosses cell membranes and is a powerful antioxidant. Melatonin reduces oxidative stress, maintains membrane stability, preserves water relations, and induces antioxidant enzyme activities in Alstroemeria cut flowers. One possible mechanism of melatonin in prolonging the vase life is by the postharvest decrease in the activity of polyphenol oxidase. Likewise, putrescine, a polyamine, also delays senescence in various plant species by impending ethylene synthesis and cellular membrane stabilization. These results are in agreement with earlier reports of beneficial effects of polyamines in extending vase life of cut flowers. Accordingly, this paper underlines the potential use of melatonin and putrescine as promising treatments for improving postharvest quality and extending vase life in Alstroemeria cut flowers. The knowledge of these pathways in the physiology of flower senescence, which includes importance on a practical sense, may thus lead to improvements in the marketability and consumer acceptance of cut flowers. Further studies will be on a more detailed description at the molecular level, in view of optimization of the treatment protocols that extend the longevity and quality of cut flowers as much as possible under various environmental conditions.

## CRediT authorship contribution statement

**Negin Hosseini:** Methodology. **Zohreh Jabbarzadeh:** Writing – original draft, Supervision, Software, Methodology, Conceptualization. **Jafar Amiri:** Writing – review & editing, Formal analysis.

## Ethics approval and consent to participate

Not applicable.

## Consent for publication

Not applicable.

## Data availability

The datasets used in this paper are available from the first author on reasonable request.

## Funding

No funding was received for this work

## Declaration of competing interest

The authors declare that they have no known competing financial interests or personal relationships that could have appeared to influence the work reported in this paper.
